# Molecular Characterization of Genome Sequences of Beak and Feather Disease Virus from the Australian Twenty-Eight Parrot (*Barnardius zonarius semitorquatus*)

**DOI:** 10.1128/genomeA.01255-14

**Published:** 2014-12-04

**Authors:** Subir Sarker, Shubhagata Das, Seyed A. Ghorashi, Jade K. Forwood, Shane R. Raidal

**Affiliations:** a School of Animal and Veterinary Sciences, Charles Sturt University, Wagga Wagga, New South Wales, Australia; b School of Biomedical Sciences, Charles Sturt University, Wagga Wagga, New South Wales, Australia; c Graham Centre for Agricultural Innovation, Wagga Wagga, New South Wales, Australia

## Abstract

Three complete genomes of beak and feather disease virus (BFDV) were recovered from wild twenty-eight parrots (*Polytelis anthopeplus monarchoides*). The genomes consisted of 1,996 bp with 1,934 identical sites and a typically content stem-loop structure between ORF1 and ORF2. This is the first report of BFDV infection as well as the complete genome sequences for this host species globally.

## GENOME ANNOUNCEMENT

Psittacine beak and feather disease (PBFD) is a chronic immunosuppressive and often fatal viral infectious threat for both the wild and captive psittacine bird species globally (1–3). Its recent discovery in critically endangered orange-bellied parrots has raised concerns for the conservation of native parrots, all of which are threatened or endangered (4, 5). All *Psittaciformes* are considered susceptible to infection since it has been reported in more than 60 species of cockatoos and parrots (3, 6–9). The etiological agent of the disease—beak and feather disease virus (BFDV), a compact circular, ambisense single-stranded DNA (ssDNA) virus belonging to the genus *Circovirus* in the family of *Circoviridae* (10, 11)—is perhaps the smallest and simplest pathogen known to infect vertebrates. Here, we report the molecular characterization of BFDV genome from three wild Australian ringneck parrots (commonly known as the twenty-eight parrot) in Western Australia.

The BFDV viral genomes were amplified from feather samples collected from three wild ringneck parrots in 1996 and stored at −20° C (sample numbers 96-B13, 96-B14, and 96-B15; GPS location: −32.533825°S, 115.5026522°E; −32.1318305°S, 116.029216°E; and −32.65904° S, 116.1297° E, respectively), and the genomic DNA was extracted using established protocols (12). Amplification of the complete genome sequences was carried out using established published protocols (13, 14). Briefly, the optimized reaction mixture contained 3 µl extracted genomic DNA, 2.5 µl of 10× High-Fidelity PCR buffer (Invitrogen), 1 µl of 25 µM of each primer, 1 µl of 50 mM MgSO_4_, 4 µl of 1.25 mM deoxynucleoside triphosphates (dNTPs), 1 U of platinum *Taq* High-Fidelity DNA polymerase (Invitrogen), and distilled water added for a final volume of 25 µl. The optimized PCR conditions were as follows: 95°C for 3 min, followed by 40 cycles of 95°C for 30 s, 57°C for 45 s, 68°C for 2 min, and finally 68°C for 5 min. Amplified PCR products were TA-cloned into pGEM-T vector (Promega) and sequenced at AGRF Ltd. (Sydney, Australia). The sequenced contigs were assembled and the entire BFDV genome was constructed using Geneious software (version 6.1.8).

The three complete genomes of BFDV from the twenty-eight parrot consist of 1,996 bp with a G+C content of 53.2%. The genome has the same basic structure as other BFDV genomes, which includes two major bidirectional transcribed open reading frames (ORFs). A preliminary BLASTn (15) analysis of the assembled sequences showed a significant (>97%) pairwise nucleotide match to a BFDV genome from the red-tailed black cockatoo (KF385399). The newly amplified BFDV genomes shared >97.0% pairwise nucleotide identity with each other, and 84.3–97.4% nucleotide sequence homology with other BFDV genomes. Consequently, based on BLASTn (15) and BLASTp (16) analyses, ORF2 encoding the capsid protein was more diverse than the virion strand encoding a replication associated protein (ORF1). The identical sites were significantly lower in the ORF2 (693 sites; 92.4%) than the ORF1 (868 sites; 99.8%).

This study highlights the evidence of BFDV infection for the first time in Australian ringneck parrots, which may provide novel insights into the viral evolution and conservation of this host species.

### Nucleotide sequence accession numbers.

The three complete genomes of BFDV have been deposited at DDBJ/ENA/GenBank under the accession numbers KF688548, KF688549, and KF688550. The versions described in this paper are the first versions.
