# Erratum for Sarker et al., Molecular Characterization of Genome Sequences of Beak and Feather Disease Virus from the Australian Twenty-Eight Parrot (Barnardius zonarius semitorquatus)

**DOI:** 10.1128/genomeA.01546-14

**Published:** 2015-01-22

**Authors:** Subir Sarker, Shubhagata Das, Seyed A. Ghorashi, Jade K. Forwood, Shane R. Raidal

**Affiliations:** a School of Animal and Veterinary Sciences, Charles Sturt University, Wagga Wagga, New South Wales, Australia; b School of Biomedical Sciences, Charles Sturt University, Wagga Wagga, New South Wales, Australia; c Graham Centre for Agricultural Innovation, Wagga Wagga, New South Wales, Australia

## ERRATUM

Volume 2, no. 6, e01255-14, 2014. Page 1: The scientific name of the twenty-eight parrot should read *Barnardius zonarius semitorquatus* in the abstract.

